# Characteristics of Gouda cheese supplemented with fruit liquors

**DOI:** 10.1186/s40781-015-0048-2

**Published:** 2015-03-25

**Authors:** Hee Young Choi, Chul Ju Yang, Kap Seong Choi, Inhyu Bae

**Affiliations:** Imsil Institute of Cheese Science, Imsil-Gun, 566-700 Korea; Department of Animal Science and Technology, Sunchon National University, 255Jungang Ro, Suncheon, Jeonnam 540-950 Republic of Korea; Department of Food Science and Technology, Sunchon National University, 255Jungang Ro, Suncheon, Jeonnam 540-950 Republic of Korea

**Keywords:** Gouda cheese, Fruit liquor, Ripening, Flavonoids

## Abstract

This study was conducted in order to evaluate the quality characteristics of Gouda cheeses supplemented with fruit liquor (*Prunusmume* or *Cornus officinalis*). Fruit liquor was supplemented to Gouda cheeses during preparation. Changes in chemical composition, lactic acid bacterial population, pH, water-soluble nitrogen, sensory characteristics, and proteolysis were monitored in the prepared ripened cheese. The electrophoresis patterns of cheese proteins, fruit liquor functional component concentrations, and the flavonoid content of the cheeses were also determined. The addition of fruit liquor did not affect (*p*> 0.05) the appearance or sensory characteristics of the cheeses. Higher amounts of crude ash, mineral, and flavonoids (*p*< 0.05) were observed in the liquor supplemented cheese than in the control cheese. Findings from this study suggest that wine supplemented Gouda could provide additional nutrients while maintaining flavor and quality.

## Background

Gouda cheese originated in the Netherlands, but similar varieties are now produced worldwide from pasteurized cow’s milk, which is acidified by a mesophilic starter containing citrate-positive bacteria. The inside diameter of an average Gouda wheel measures approximately 25.4 cm, with a thickness of 16.5 cm. The percentage of water in this particular cheese varies from 41.25 to 45.43%, with an average of 43.5% (Charles, [1]).

Four-year-old specialty Gouda is riddled with small holes formed by gases and subsequently filled by amino-acid crystals [2]. Despite the minimal share (0.24%) of wine in the Korean alcoholic beverage market, wine is the one of the fastest growing commodities among alcoholic beverages and liquors.

*Prunusmume* Sieb. Et Zucc (Measil), which is found in Continental Southeast Asia, Korea, China, and Japan, and distributed only in temperate regions of various Oriental countries is a unique plant variety [3]. *P. mume* fruit are processed commercially into pickled products, juices, or liquors in Japan [4]. Consumption has recently increased, and as a result, it is necessary to re-evaluate their nutritional value and their value as a healthy food. Some of the polysaccharide fractions in *P. mume* exhibit various biological activities, including the mutagenicactivation of the alternative pathway of the complement complex, the activation of clot formation in human plasma, andantioxidant activity and free radical scavenging in methanol aqueous extracts of 100 plants [5].

*Cornus officinalis* Siet. Et Zucc (Sansuyu) fruit can treat symptoms associated with liver or kidney deficiencies, diabetes, and uterine bleeding [6]. Park *et al.* [7] reported that C. *officinalis* is an excellent antioxidant. Tannins, galloylatedglycosides, gallotannins, organic acids, and furan derivatives have been reported from fruits of *C. officinalis*. In fact, triterpenoids, mainly oleanolic acid, ursolic acid, and the glycosides derived from these substances, have been identified in *C. officinalis* fruit [8].

In this study, we examined the quality characteristics of Gouda cheese supplemented with fruit liquor made with *Prunusmume* (Measil) or *Cornus officinalis* (Sansuyu) in order to investigate the functional properties of the supplemented Gouda cheese.

## Methods

### Fruit liquor

Fruit liquor made of *Prunusmume* (Measil, PM) or *Cornus officinalis* (Sansuyu, CO) was purchased at local markets in Suncheon, Korea.

### Gouda cheese supplemented with fruit liquor

Gouda cheese was made as described by Mistry and Pulgar [9] and Hill [10] with some modifications. Raw milk was obtained from the dairy farm of Sunchon National University (Suncheon, Republic of Korea). PM or CO fruit liquor was added to fresh raw milk at 4.0% of total milk volume, pasteurized at 63°C for 30 min, and cooled to 32°C. The milk was held in a vat, inoculated with Probat 505 DVS (Direct Vat Set) starter (*Lactococcuslactis* subsp. *lactis*, *L. lactis* subsp. *cremoris, L. lactis* subsp. *Lactis* biovar. *diacetylactis, Leuconostoc mesenteroides* subsp. *cremoris;* Danisco, Copenhagen, Denmark) at 2.5 g/100 kg, incubated for 45 min, and a solution of calcium chloride (20 mL/ 100 kg; CALCIO, Danisco, Copenhagen, Denmark) was added. After incubation, rennet (Christian Hansen, Hørsholm, Denmark) curd cut into cubes 5.0 – 7.0 mm^3^ in size was added (19 mL/100 kg) in order to induce the coagulation of the milk. Curds were formed, and the curd was cut into cubes 10 mm^3^ in size. The cut curd was allowed to settle for 4 min to heal, and then was stirred at 32°C for 30 min. The whey (30%) was discarded, and the same volume of hot water (~75°C) was added to raise the vat temperature from 32 to 38°C. After agitation for 1 h, half of the whey was removed, and the curd was pressurized at 1 times the weight of the curd in a vat with half of the remaining whey. The pressure starts at 1 times the weight of the curd, but after 1 hour the pressure was increased to 2 times the curd weight for 1 h. When the pH of the cheese reached 5.1-5.2, the curd was immersed in a 20% NaCl (pH 5.1 ~ 5.2) solution for 8 h/kg and matured at 14°C with 90 ~ 95% relative humidity (R/H.) for 15 weeks.

### Enumeration of lactic acid bacteria (LAB) and pH measurement

The number of LAB was monitored every three weeks during ripening. Samples were mixed with sterilized saline and cheese samples at a ratio of 2:1 and homogenized (M. Zipper GmbH, Germany) at maximum speed for 2 min. According to the method of Richardson *et al.* [11], 1.0 mL of homogenized sample was diluted aseptically with sterilized saline and spread on MRS agar (BBL/Difco, USA). The MRS agar plates were incubated at 37°C for 48 h, and the colonies were counted. The cheese samples were homogenized with sterile saline at a1:2 ratio and measured using a pH meter (IQ Scientific Instruments Inc, San Diego, USA).

### Water-soluble nitrogen (WSN)

In order to measure the total protein decomposition level during cheese ripening, change in WSN was measured according to the method described in Bütikofer [12].

The sample used for measurement of the change in WSN was homogenized and centrifuged (Supra 25 K, Hanil Science Industrial, Korea) as described in the pH measurement section, and the filtrate (Whatman No.2) was then colored using the method described in Hull [13]. Next, the content of WSN was measured at 570 nm using a UV- Spectrophotometer (Smart Plus Spectrophotometer Co., Korea). The content of a nitrogenous compound was calculated according to a linear regression equation obtained by making tyrosine the standard substance.

### Sodium Dodecyl Sulfate (SDS)-Gel electrophoresis of casein

Polyacrylamide gel electrophoresis of a casein sample was performed using the method described in Laemmli [14]. The cheese sample was prepared by adding 6 ml of TCA (12.0%) to the cheese (0.3 g), followed by sedimentation and filtration with a filter paper (Whatman No. 42). The filtrates were dissolved with 0.076 M Tris-citrate buffer (pH 9.0) at *ca*. 30 mg/mL. 40 μL of solution dialyzed with electrode buffer for 48 h were boiled in the SDS sample buffer (×5) for 3 min to denature the proteins. The boiled samples were carefully loaded up to 10 μL, the current was adjusted to 30 mA for a 1.5-mm-thick gel, and then electrophoresis was started. The gel was prepared at a pH of 8.8 and 15% polyacrylamide. Whole casein (Bio-Rad Laboratories, Hercules, CA, USA) was used as a standard protein marker. After the completion of electrophoresis, the gel was stained with Coomassie brilliant blue gel stain and then bleached and photographed.

### Determination of polyphenol content

To determine the polyphenol content in the cheeses, extracts (100 mL) were obtained by adding ethanol to homogenized samples. Extracts (5 mL) obtained after the first 20 mL had been discarded were mixed with 5.0 mL of ferrous tartrate stock solution (100 mg FeSO_4_ · 7H_2_O + 500 mg/100 mL H_2_O Rochelle salt), and the pH was adjusted to 7.5 with 0.066 M Na_2_HPO_4_ · 2H_2_O and 0.066 M KH_2_PO_4_. Then, the absorbance of the treated extracts was assessed at 540 nm using a spectrophotometer.

### Proximate composition analysis

AOAC methods [15] were used to determine the moisture, protein, and fat content in the cheeses.

### Analysis of thiobarbituric acid (TBA)

Cheeses that had completely ripened were stored in a refrigerator for 0–5 weeks for measurement of rancidity. Cheese samples (20 g) were supplemented to 50 mL of 2 M phosphoric acid with 20% TCA to obtain extracts. Extract residues were diluted in 40 mL of distilled water, homogenized, and filtered with Whatman #1 paper. The filtrates (5.0 mL) were mixed with 2-thiobarbituric acid, maintained for 15 h at room temperature, and the absorbance was read at 530 nm using a spectrophotometer (Model 20D’, Milton Roy, Ivyland, PA, USA).

### Sensory evaluation

The cheeses were tested in triplicate by a highly trained panel of seven assessors at Kansas State University’s Sensory Analysis Center, KS, USA. A scale of 0–15 was used, and the answers to 27 questions were evaluated [16,17].

### Statistical analyses

Data were analyzed by ANOVA using SAS software (SAS Institute, Cary, NC, USA). Fisher’s least significant difference test was used for the determination of differences in chemical composition and polyphenol content between treatments. Significance was detected at the 95% confidence level (*p* ≤ 0.05).

## Results and discussion

### Appearance

Gouda cheeses with fruit liquor were similar in color to the control cheese. The ripening period was faster in cheese supplemented with fruit liquor than in the control cheese, and texture, hardening, and density were greater in cheese supplemented with fruit liquor than in the control cheese. It is assumed that the addition of fruit liquor aided in the natural ripening and texture of the Gouda cheese.

### Composition

The chemical composition and mineral content of the Gouda cheese supplemented with traditional fruit liquor are shown in Table 1. Moisture and crude protein content did not differ between the treatments. Cheese supplemented with PM and CO fruit liquor contained a higher amount of crude ash and crude fat than the control cheese. In addition, cheese supplemented with PM fruit liquor contained a higher amount of crude ash than the CO-supplemented cheese and the control cheese. In agreement with the current study, Song *et al.* [18] reported that PM fruit liquor increased crude ash from 0.57% to 0.69%. Cheese supplemented with CO fruit liquor contained 5.6% crude fat, which was greater than that of the control cheese [19].

**Table 1:** **Chemical and Mineral composition of the Gouda cheese supplemented with fruit liquors**

***Component***	***Cheeses supplemented with***		
**(%)**	**Nothing**	**PM fruit liquor**	**CO fruit liquor**
Moisture	38.05 ± 0.13^ns^	38.103 ± 0.05^ns^	37.45 ± 0.92^ns^
Crude ash	3.23 ± 0.02^b^	3.70 ± 0.83^a^	3.41 ± 1.01^b^
Crude protein	26.50 ± 0.21^ns^	27.35 ± 0.94^ns^	26.63 ± 0.25^ns^
Crude fat	31.22 ± 0.31^b^	31.72 ± 0.44^b^	33.52 ± 1.34^a^
Fe	2.10 ± 0.27^c^	3.75 ± 0.02^a^	2.75 ± 0.85^b^
Mg	6.80 ± 0.30^c^	21.01 ± 0.05^a^	17.18 ± 0.08^b^
Ca	559.96 ± 22.87^a^	222.024 ± 9.01^c^	252.86^g^ ± 20.05^b^

Means with the same superscripts in the same row are not significantly different (p > 0.05).

The current study found similar results with regard to the crude fat content. The mineral composition of Gouda cheese supplemented with fruit liquor is shown in Table 2. Iron and magnesium content in Gouda cheese supplemented with PM and CO fruit liquor were slightly higher than in the control cheese. However, calcium content was two-fold higher in the control cheese than in cheese supplemented with wine.

**Table 2:** **Changes in pH and LAB content of fruit liquors supplemented Gouda Cheese during ripening**

***Component***	***Day***	***Cheeses supplemented with***		
		**Nothing**	**PM fruit liquor**	**CO fruit liquor**
pH	0	5.24	5.24	5.24
	3	5.13	5.28	5.11
	6	5.11	5.22	5.24
	9	5.28	5.29	5.22
	12	5.24	5.31	5.32
	15	5.15	5.25	5.39
LAB	0	9.98	10.15	10.17
	3	9.43	9.15	10.11
	6	9.10	9.40	9.60
	9	8.35	8.30	9.13
	12	7.70	8.16	8.70
	15	8.20	8.25	8.84

### Lactic acid bacteria (LAB)

The population of LAB was slightly higher in the cheese supplemented with fruit liquor than in the control cheese (Table 2). The cheese supplemented with CO fruit liquor contained significantly higher numbers of LAB after the third day than the control cheese. Cheese supplemented with traditional fruit liquors not only had accelerated cheese ripening but also produced a unique taste during long-term ripening, which may be attributed to the fact that the fruit liquors contained bioactive substances.

### pH

The pH values of the cheeses during ripening are shown in Table 2. The pH values (5.11-5.39) of the cheeses supplemented with traditional fruit liquor were slightly lower than those of the control cheeses. After the fermentation period, the pH of cheese supplemented with fruit liquor was 5.2-5.3 and that of the control cheese was 5.1-5.4, which is generally an optimal pH of Gouda cheese for maintenance of a pleasant, mild taste. Cheese supplemented with fruit liquor had slightly increased pH values due to lactic acid formation from the degradation of lactose in the cheese. These results are supported by those of Lawrence *et al.* [20], who found that this process occurred by the catabolism of residual lactic acid.

### Water-soluble nitrogen (WSN)

The WSN content in the cheeses changed with the progress of ripening (Table 3). Sallami *et al.* [21] reported that a protease produced by LAB in cheeses caused constant protein degradation. The WSN content of the cheese supplemented with traditional fruit liquor were slightly higher than those of the control cheese throughout the ripening period. The WSN content increased more between six and 12 weeks than at the beginning of ripening. An increase in the amount of nitrogen compounds was observed with ripening in all cheeses. Morgan *et al.* [22] reported that most nitrogen compounds of ripening cheeses were affected by products of LAB, which is similar to the current results.

**Table 3:** **Changes in the water-soluble nitrogen (WSN) content of fruits liquor-supplemented Gouda cheese during ripening**

***Aging time (weeks)***	***Cheese supplemented with***		
	**Nothing**	**PM fruit liquor**	**CO fruit liquor**
0	11.91	7.30	5.78
3	12.69	23.67	24.84
6	23.10	30.32	32.92
9	40.58	43.61	41.70
12	42.27	51.38	48.45
15	45.19	47.78	49.13

### Casein proteolysis

**Figure 1:**
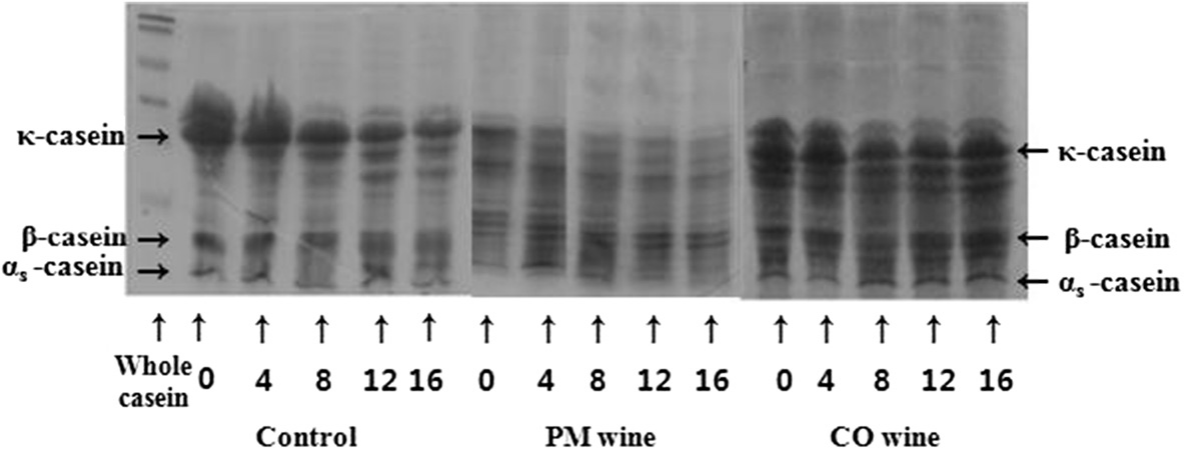
**Proteolytic activity of Gouda cheese supplemented with fruits liquors during ripening.** SDS-standard, PM liquor, and CO liquor.

The degradation of protein during ripening of Gouda supplemented with fruit liquor is shown in Figure 1. Laemmli [14] analyzed samples over 16 weeks, collected every four weeks, using SDS-PAGE. Due to proteolytic activity, protein degradation was slightly higher in cheese supplemented with fruit liquor than in the control cheese.

### Determination of polyphenols, anthocyanin, and flavonoid content

The polyphenol, anthocyanin, and flavonoid contents in the PM and CO fruit liquors are shown in Tables 4 and 5. The polyphenol content (889 mg/100 g) was higher than the anthocyanin (0.18 mg /100 g) or flavonoid (30.5 mg/100 g) contents in PM fruit liquors. The recovery rate of polyphenols (1,034 mg/100 g) from CO fruit liquor cheeses increased with longer ripening periods. The flavonoid content increased, from 301.1 mg/100 g in week 0 to 503.8 mg/100 g at week 15; the flavonoid content increased until week 9 and then showed little change thereafter. These results indicate that the flavonoid content may be useful as an indicator of protein degradation in PM fruit liquor cheeses.

**Table 4:** **Polyphenol, anthocyanin, and flavonoid contents in fruit liquors**

***Component***	***Cheeses supplemented with***		
	**Nothing**	**PM fruit liquor**	**CO fruit liquor**
*Polyphenols*	-	889 ± 0.2^a^	1.304 ± 0.1^a^
*Anthocyanins*	-	0.18 ± 0.0^a^	0.64 ± 0.16^a^
*Flavonoids*	-	30.5 ± 0.1^a^	15.1 ± 0.2^a^

Means with the same superscripts in the same row are not significantly different (p > 0.05).

**Table 5:** **Change in flavonoid content (mg/100 g cheese) of liquor-supplemented Gouda cheese during ripening**

***Aging time (weeks)***	***Cheese supplemented with***		
	**Nothing**	**PM fruit liquor**	**CO fruit liquor**
0	25.1 ± 0.02^a^	301.1 ± 0.22^a^	280.5 ± 0.43^a^
6	26.5 ± 0.14^a^	420.9 ± 0.47^b^	431.2 ± 0.17^b^
9	26.5 ± 0.11^a^	508.3 ± 0.23^c^	511.5 ± 0.15^c^
15	26.8 ± 0.52^a^	503.8 ± 0.11^c^	510.9 ± 0.32^c^

Means with the same superscripts in the same row are not significantly different (p > 0.05).

### Analysis of thiobarbituric acid

Thiobarbituric acid (TBA) values were measured during Ripening of cheeses at refrigerated temperatures for nine months (Table 6). TBA values in the stored cheeses did not differ until 2–3 months among control and cheeses supplemented with fruit liquor. However, after three months, TBA values were approximately 2–6 times higher in control cheeses compared with cheeses supplemented with fruit liquors. In addition, the oxidation of cheese supplemented with fruit liquors did not progress actively.

**Table 6:** **Changes in thiobarbituric acid (TBA) values of liquor-supplemented Gouda cheese during the Storage period**

***Storage time (Months)***	***Cheese supplemented with***		
	**Nothing**	**PM fruit liquor**	**CO fruit liquor**
1	10.10	4.56	4.85
2	13.50	4.99	8.95
3	10.22	1.12	3.84
4	25.80	2.11	7.60
5	35.10	4.21	12.10
6	64.20	1.49	18.50
7	61.44	3.86	20.10
8	59.32	3.64	17.80
9	60.88	3.95	17.88

### Sensory evaluation

**Figure 2:**
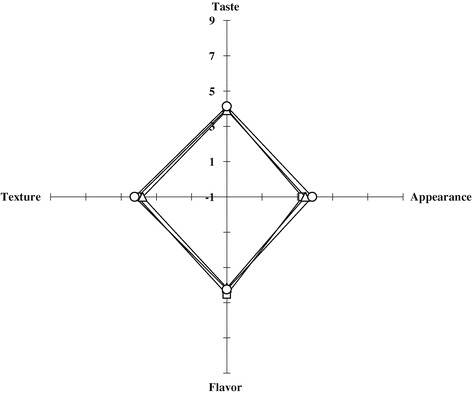
**Sensory evaluation of Gouda cheese supplemented with fruits liquor.**

The results of the sensory evaluations of taste, appearance, flavor, and texture of Gouda cheese supplemented with fruit liquors are shown in Figure 2. Most of the cheeses supplemented with traditional fruit liquors were rated lower than the control cheeses. In fact, CO fruit liquor cheese was rated below all of the other cheeses. Among the experimental cheeses, the highest flavor preference was found in cheese supplemented with PM fruit liquor. Additional studies will be necessary in order to further examine the effects of fruit liquor and the ripening conditions of the cheeses.

## Conclusions

Our results from a compositional analysis of Gouda cheese supplemented with fruit liquors (PM or CO) showed no significant differences in moisture and crude protein, but both were significantly higher in crude mineral and crude fat than the control cheese. Populations of lactic acid bacteria and WSN in cheese supplemented with wine were higher, and pH values were lower, than those of the control cheese. SDS-PAGE revealed additional bands found in cheese supplemented with fruit liquors versus the control cheese, indicating a higher rate of proteolysis in cheese supplemented with fruit liquor. In the case of flavonoids, cheese supplemented with PM fruit liquor tended to have an increasedrecoveryrate with ripening period and TBA levels. Sensory characteristics were lower in cheeses supplemented with fruit liquor than in the control cheese. Further studies on the functional composition of the cheeses and new manufacturing methods will be needed.
